# Gearing up for survival - HSP-containing granules accumulate in quiescent cells and promote survival

**DOI:** 10.15698/mic2016.03.481

**Published:** 2016-03-06

**Authors:** Ruofan Yu, Weiwei Dang

**Affiliations:** 1 Huffington Center on Aging, Department of Molecular and Human Genetics, Baylor College of Medicine, Houston, TX 77030, USA.

**Keywords:** budding yeast, stationary phase, chronological aging, quiescence, cytoplasmic granules, Hsp42

The budding yeast *Saccharomyces cerevisiae* proliferates in a logarithmic fashion when growing in glucose-containing rich media. When all carbon sources in the environment are depleted, the yeast cells enter the stationary phase. There are two types of stationary cells: the quiescent (Q) cells, which temporarily cease dividing before nutrition replenishes, and the non- quiescent (NQ) cells that keep proliferating 1. Both physical and biochemical differences exist between the Q and NQ cell types. For example, Q cells have a higher density and the mRNA and protein profiles in the two cell types are also profoundly different 2,3. Apart from these features, Q cells have been reported to have higher resistance to stress and a longer chronological lifespan.

Cells within the stationary phase are commonly observed to have granule structures, containing stress response-related factors 4 and may thus play a role in the elevated survival ability of Q cells. In this issue, Lee *et al*. reports that distinct granules are formed in Q and NQ cells, which determines their respective cell fates 5. The authors asked whether different kinds of granules were distributed uniformly between Q and NQ cells, and found that most granule structures are enriched in NQ cells with the exception of Hsp42-associated stationary phase granules (Hsp42-SPGs) (Fig. 1). Using a mutated form of luciferase that misfolds upon heat shock, they further demonstrated that Hsp42-SPGs contributes to stress response of Q cells by facilitating clearance of protein aggregates, which is consistent with the reported function of Hsp42 to prevent unspecific protein aggregation 6. Lee *et al*. also proved that the Q cells reenter the mitotic cycle more quickly after nutrition replenishment 5, which further establishes the phenotypical difference between the two cell types (Fig. 1). Since Q and NQ cells come from the same clone, their formation has been proposed to be a process of cell differentiation 3. Consistent with this idea, this paper shows that NQ cells are indeed committed to their cell fate: most NQ cells remain non-quiescent even after re-entering the cell cycle (Fig. 1). This study, coupled with other research on yeast quiescence, will likely provide valuable insight into cell differentiation in higher eukaryotes.

**Figure 1:**
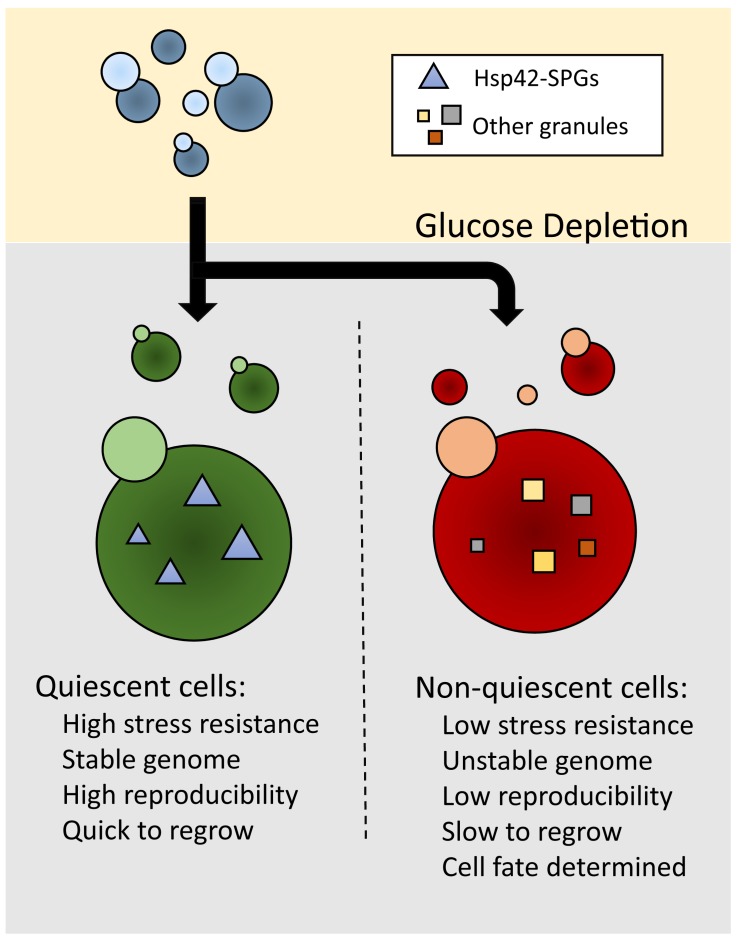
FIGURE 1: Hsp42-associated stationary phase granules (Hsp42-SPGs) confer features for quiescent cells (Q) that are distinct from non-quiescent cells (NQ).

A key question remains to be addressed in the field: what determines a cell to become quiescent or non-quiescent ? The molecular pathways that regulate this cell-fate commitment remain elusive. Davidson et al. 7 suggested that differentiation depends on epigenetic changes rather than DNA mutation. This is probable since acetyl-CoA metabolism changes upon entering the stationary phase 8, and acetyl- CoA, being the major acetyl group donor, has a substantial impact on protein acetylation. While some explanation is still required for the drastic difference between the two cell types, the molecular mechanisms linking environmental changes with quiescence will be an important step in the long-lasting study of the relationship between environment and aging.

## References

[1] Gray JV, Petsko GA, Johnston GC, Ringe D, Singer RA, Werner-Washburne M (2004). “Sleeping beauty”: quiescence in Saccharomyces
cerevisiae.. Microbiol Mol Biol Rev.

[2] Aragon AD, Rodriguez AL, Meirelles O, Roy S, Davidson GS, Tapia PH, Allen C, Joe R, Benn D, Werner-Washburne M (2008). Characterization of differentiated quiescent and nonquiescent
cells in yeast stationary-phase cultures.. Mol Biol Cell.

[3] Palková Z, Wilkinson D, Váchová L (2014). Aging and differentiation in yeast populations: elders with
different properties and functions.. FEMS Yeast Res.

[4] Haslbeck M, Franzmann T, Weinfurtner D, Buchner J (2005). Some like it hot: the structure and function of small heat-shock
proteins.. Nat Struct Mol Biol.

[5] Hsin-Yi Lee, Kuo-Yu Cheng, Jung-Chi Chao, Jun-Yi Leu (2016). Differentiated cytoplasmic granule formation in quiescent and
non-quiescent cells upon chronological aging.. Microbial Cell.

[6] Shah KH, Zhang B, Ramachandran V, Herman PK (2013). Processing body and stress granule assembly occur by independent
and differentially regulated pathways in Saccharomyces
cerevisiae.. Genetics.

[7] Davidson GS, Joe RM, Roy S, Meirelles O, Allen CP, Wilson MR, Tapia PH, Manzanilla EE, Dodson AE, Chakraborty S, Carter M, Young S, Edwards B, Sklar L, Werner-Washburne M (2011). The proteomics of quiescent and nonquiescent cell differentiation
in yeast stationary-phase cultures.. Mol Biol Cell.

[8] Dos Santos MM, Gombert AK, Christensen B, Olsson L, Nielsen J (2003). Identification of in vivo enzyme activities in the cometabolism
of glucose and acetate by Saccharomyces cerevisiae by using 13C-labeled
substrates.. Eukaryotic Cell.

